# Reduction of butyrate- and methane-producing microorganisms in patients with Irritable Bowel Syndrome

**DOI:** 10.1038/srep12693

**Published:** 2015-08-04

**Authors:** Marta Pozuelo, Suchita Panda, Alba Santiago, Sara Mendez, Anna Accarino, Javier Santos, Francisco Guarner, Fernando Azpiroz, Chaysavanh Manichanh

**Affiliations:** 1 Digestive System Research Unit, Vall d’Hebron Research Institute, Passeig Vall d’Hebron 119-129, Barcelona 08035, Spain; 2 Digestive Unit, University Hospital Vall d’Hebron, Passeig Vall d’Hebron 119-129, Barcelona 08035, Spain; 3 Centro de Investigacion Biomedica en Red en el Área tematica de Enfermedades Heptaticas y Digestivas, CIBERehd, Instituto de Salud Carlos III, Madrid, Spain

## Abstract

The pathophysiology of irritable bowel syndrome (IBS) remains unclear. Here we investigated the microbiome of a large cohort of patients to identify specific signatures for IBS subtypes. We examined the microbiome of 113 patients with IBS and 66 healthy controls. A subset of these participants provided two samples one month apart. We analyzed a total of 273 fecal samples, generating more than 20 million 16S rRNA sequences. In patients with IBS, a significantly lower microbial diversity was associated with a lower relative abundance of butyrate-producing bacteria (P = 0.002; q < 0.06), in particular in patients with IBS-D and IBS-M. IBS patients who did not receive any treatment harboured a lower abundance of Methanobacteria compared to healthy controls (P = 0.005; q = 0.05). Furthermore, significant correlations were observed between several bacterial taxa and sensation of flatulence and abdominal pain (P < 0.05). Altogether, our findings showed that IBS-M and IBS-D patients are characterized by a reduction of butyrate producing bacteria, known to improve intestinal barrier function, and a reduction of methane producing microorganisms a major mechanism of hydrogen disposal in the human colon, which could explain excess of abdominal gas in IBS.

A recent review reported that irritable bowel syndrome (IBS) affects around 11% of the population worldwide, with the lowest prevalence occurring in South Asia (7%) and the highest in South America (21%)^1^. However, it should be noted that prevalence-reporting rates are subject to the diagnostic criteria used. Although most clinicians use the Rome criteria for this purpose^2^, the lack of biological markers leads them to frequently resort to other clinical findings, such as bloating and psychological stress.

The pathophysiological mechanisms underlying IBS are not fully known. Abnormal gastrointestinal (GI) motility, visceral hypersensitivity, altered brain-gut function, low-grade inflammation, and psychosocial disturbance, have been recognized in different subsets of patients. In addition, the onset of IBS following infective gastroenteritis and the involvement of small bowel bacterial overgrowth (SIBO), suggest that gut microbes play a role in at least some of the mechanisms leading to IBS. Fermentation of polysaccharides by colonic microorganisms can produce a number of by-products (gases- H2 and CH4 (methane) and short-chain fatty acids (SCFAs) such as acetate, propionate and butyrate) that may have important implications in bowel movement and epithelial permeability^3^,^4^. The current working hypothesis is that an abnormal microbial composition activates mucosal innate immune responses, which increase epithelial permeability, activate nociceptive sensory pathways, and dysregulate the enteric nervous system^5^.

Supplementary Table S1 reports the methods and main results gathered from 24 studies on IBS and microbiome using culture-independent techniques. Over the past ten years, most of these studies have used 16S rRNA gene (16S) surveys through quantitative specific polymerase chain reaction (qPCR), denaturing gradient gel electrophoresis (DGGE), terminal restriction fragment length polymorphism (T-RFLP), fluorescent *in situ* hybridization (FISH) or cloning, and Sanger sequencing to characterize the microbiome of patients with IBS. Only since 2011 have a few studies used high-throughput techniques, such as 16S phylogenetic microarray, 16S and shotgun pyrosequencing, and metatranscriptomics.

Results from those studies showed several common trends, as well as inconsistencies in the microbial signatures of patients with IBS or subtypes of this condition. Among the trends, patients show dysbiotic microbiota, which can be characterized at various phylogenetic levels. At the phylum level, a higher proportion of Proteobacteria^6^,^7^ has been reported in patients compared to healthy controls. At the genus level, a higher count or proportion of *Veillonella*^8^,^9^,^10^, *Lactobacillus*^9^,^10^,^11^,^12^ and *Ruminococcus*^7^,^8^,^12^,^13^, has been associated with IBS. In contrast, a lower count of *Bifidobacterium*^10^,^13^,^14^,^15^, *Faecalibacterium*^13^,^16^, and methanogens^13^,^17^ was encountered in patients with this condition. Among the inconsistencies, three studies reported a higher ratio of Firmicutes/Bacteroidetes in patients^6^,^13^,^18^, while the opposite was found in one study^19^. Lower^20^ and higher^21^ counts of *Eubacterium rectal* were found in two studies. Also, it has been proposed that IBS involves a higher count of *Clostridium coccoides*^8^,^20^, while other authors reported a lower count of this species. Despite the replacement of culture methods by more powerful molecular techniques, these studies described small sample sizes, ranging from 2 to 62 IBS patients and did not consider confounding factors such as medications for IBS symptoms.

Using a high number of healthy controls (n = 66) and IBS patients (n = 113), two-time points for 94 participants, and a deep sequencing coverage of the 16S rRNA gene, we sought to determine: (a) whether dysbiosis occurs in patients with IBS and, if so, the phylogenetic level that defines it; (b) whether the three IBS subtypes can be distinguished by microbial community clustering; (c) whether microbial diversity is lower in patients compared to healthy controls and, if so, which bacteria are absent; (d) the temporal stability of the microbial community in IBS patients; (e) the effect of medication on the microbiota of IBS patients and (f) the correlation between microbiota and the patients’ symptoms.

## Results

### Sequence data description

Fecal samples were collected from 66 healthy controls and from 113 IBS patients. Of the 179 participants, 94 provided two fecal samples 1-month apart. We therefore analyzed the microbiome of a total of 273 samples, from which we produced 20.5 million high quality sequence reads of 314 bp in length, using the default Quantitative Insights Into Microbial Ecology (QIIME) pipeline parameters and a quality Phred score >20. Additionally, we removed singletons and taxa with very low abundance that could represent false positive taxa, as described in the method section. We finally recovered a total of 14 million sequences with a mean of 48,409 sequences per sample. In order to compare microbial community between samples, we normalized the sequences at 16,800 per sample (fitting to a sample with the lowest sequence number).

### Microbiota of the healthy controls

Of the 66 healthy controls, we detected 12 known phyla, 43 families, 84 genera (Supplementary Figure S1) and 2,126 operational taxonomic units (OTUs), with a mean of 629 OTUs (SD = 132) per sample. Firmicutes and Bacteroidetes accounted for 91% of the sequences, and Proteobacteria, Verrucomicrobia and Actinobacteria 4.9%. At the family level, 85% of the sequences were assigned, 65% at the genus level and only 0.6 % at the species level. This observation indicates that although databases of 16S rRNA sequences are increasing exponentially, the lack of annotation continues to be a bottleneck for the scientific community. Our findings thus revealed the predominance of Firmicutes in the proportion of sequences per subject and in the proportion of the healthy population. Two OTUs, *Faecalibacterium prausnitzii* and an unknown Ruminococcaceae, were detected in all individuals and at the two sampling points (88 samples in total), thereby indicating that these OTUs were not only highly prevalent but also stable over time. They were found at the average proportions of 5.3% and 0.23%, respectively.

### Level of dysbiosis among IBS patients

**Figure 1:**
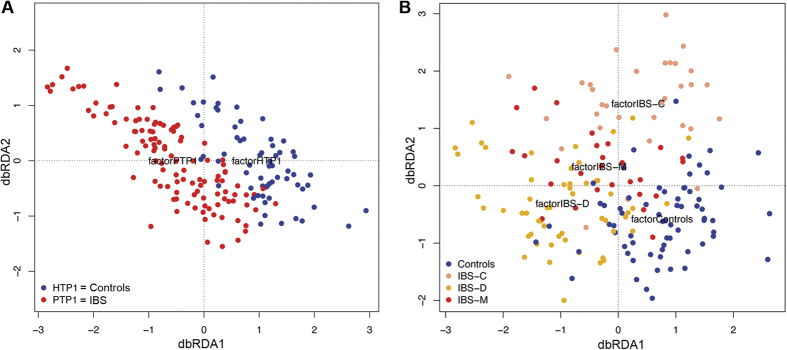
Unweighted UniFrac data redundancy analysis on the first time point samples constrained by (**A**) controls and IBS patients groups, and (**B**) constrained by the four groups of participants: controls (n = 66), IBS-C (n = 32), IBS-D (n = 54) and IBS-M (n = 27).

At a global level, the microbial communities of healthy controls and patients did not cluster separately, according to Unifrac metrics in a principal coordinate analysis (PCoA, Supplementary Figure S2). However, distance-based redundancy analysis showed that the microbiome of IBS patients, as well as that of IBS subtype patients, clustered separately from that of healthy controls (P = 0.002 and P = 0.001, respectively)(Fig. 1A,B), although only 1 and 3% of the data explained the variation observed in IBS and IBS subtypes, respectively.

**Figure 2:**
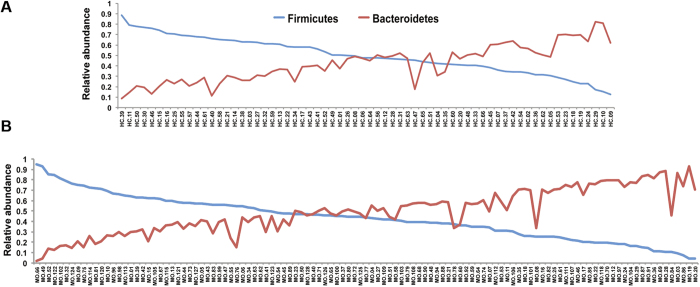
Higher relative abundance of Bacteroidetes. Proportion of Firmicutes and Bacteroidetes are plotted for each healthy subject (**A**) and for each IBS patient (**B**). Patients are characterized by a relatively higher proportion of Bacteroidetes than healthy controls (P = 0.02, q = 0.09).

**Figure 3:**
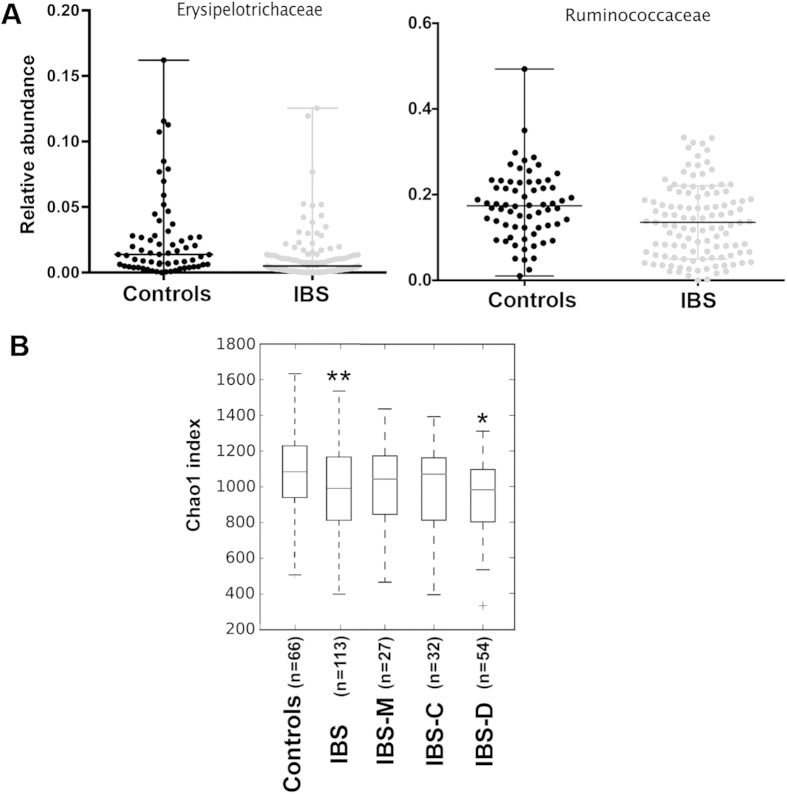
Higher relative abundance of two bacterial families and higher alpha-diversity in healthy controls compared to IBS patients. (**A**) Erysipelotrichaceae and Ruminococcaceae were found in significantly higher abundance in healthy subjects (n = 66) compared to IBS patients, regardless of their IBS subtype (Kruskal Wallis test, P = 4.7 e-5, q = 0.002 and P = 0.002, q = 0.06, respectively). The two bacterial families belong to the Firmicutes phylum. (**B**) The Chao1 index based on species-level OTUs was estimated for healthy controls, IBS, IBS-M, IBS-C and IBS-D. Significance (*P = 0.04, **P < 0.003) was determined by Monte Carlo permutations, a non-parametric test.

Using the Kruskal Wallis test to compare healthy controls (n = 66) with IBS patients (n = 113), dysbiosis was indeed present at various phylogenetic levels. At the phylum level, there is a tendency for IBS patients to harbour a higher average count of Bacteroidetes compared to healthy controls (52.6% versus 42.7%; P = 0.02, q = 0.09) and a lower count of Firmicutes (39.8% versus 49%; P = 0.02, q = 0.09). Furthermore, 41% of IBS patients (versus 50% in healthy subjects) harboured a higher relative abundance of Firmicutes than Bacteroidetes (Fig. 2). Compared to healthy controls, IBS patients also showed a lower count of Tenericutes (P = 0.004; q = 0.05). At the family level, two Firmicutes groups, Erysipelotrichaceae and Ruminococcaceae, were found significantly in a higher proportion in healthy controls than in patients (Fig. 3A) (P = 4.7 e-5, q = 0.002 and P = 0.002, q = 0.06, respectively). At the species level, one OTU from the *Lachnobacterium* genus (Lachnospiraceae, Firmicutes) differentiated healthy subjects from IBS patients (P = 5.9 e-6, q = 0.04). This OTU, which belongs to the Lachnospiraceae family and the Firmicutes phylum, was detected in 15 out of 66 controls (22%) and in only 2 out of 113 patients (1.7%) at a very low proportion of sequences, 0.0001 and 0.00002 respectively. *Faecalibacterium prausnitzii* present in all 66 healthy controls, were also detected in at least 95% of the 113 patients and did not show significant differences in abundance. This observation indicates that this bacterium is highly prevalent but probably not involved in IBS, as proposed by others^13^.

### Microbiota and IBS subtypes

**Figure 4:**
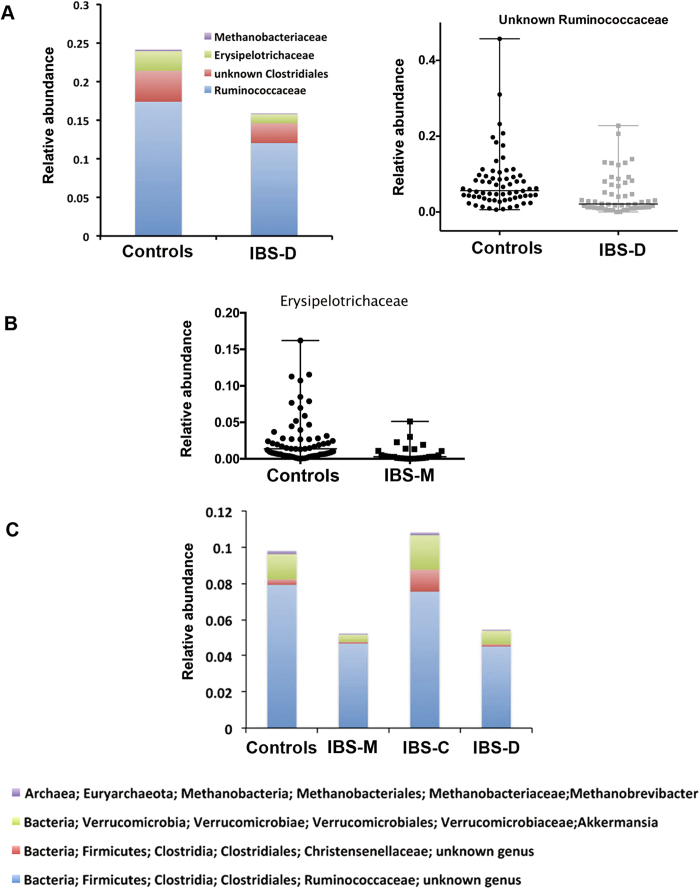
Dysbiosis at the family and genus level in IBS subtypes. (**A**) Four microbial families and one genus discriminate the 66 healthy controls from the 54 patients with IBS-D (Kruskal Wallis test, P < 0.006; q < 0.06). (**B**) One bacterial family was found in a lower proportion in the 27 IBS-M patients compared to the 66 controls (P = 0.0001, q = 0.006). (**C**) Comparing the healthy control group with the three IBS subtypes, four genera were enriched in healthy subjects and IBS-C patients compared to IBS-M and IBS-D patients (Kruskal Wallis test, P < 0.003, q ≤ 0.05).

The diversity analysis showed that, overall, patients presented a lower diversity of gut microbiota than healthy controls (P < 0.01), especially as a result of a reduction in the diversity of the IBS-D subtype (P < 0.05; Fig. 3B). This result suggests that the microbial species that contribute to maintaining homeostasis may be missing in patients with a diarrheic symptom. Therefore, we compared the microbial communities of healthy participants (n = 66) with those of each IBS subtype using the Kruskal Wallis test. Patients with IBS-D (n = 54) showed dysbiosis at various phylogenetic levels. Figure 4A shows the lower abundant groups at the family (Ruminococcaceae, unknown Clostridiales, Erysipelotrichaceae, Methanobacteriaceae; P < 0.006; q < 0.06) and genus levels (unknown Ruminococcocaceae; P = 0.0001, q = 0.009), respectively.

The microbiome of IBS-C patients did not show significant differences at any phylogenetic level with that of healthy controls. However, IBS-M patients, who alternate diarrhea and constipation, were associated with a 4.7-fold lower abundance of Erysipelotrichaceae (P = 0.0001, q = 0.006; Fig. 4B), sharing this difference with IBS-D patients. Our findings suggest that this bacterial group, which is absent in IBS-M and IBS-D, is associated with a non-diarrheic phenotype.

Comparing the healthy control group with the three IBS subtypes, we found that four genera were significantly different between healthy subjects/IBS-C patients and IBS-M/IBS-D patients. Indeed, an unknown Ruminococcaceae, an unknown Christensenellaceae, *Akkermansia*, and *Methanobrevibacter* were present in a higher proportion in healthy controls and IBS-C patients (P < 0.003, q ≤ 0.05; Fig. 4C).

### IBS patients and treatment

Regarding treatment, the inclusion criteria for all participants was only that they had not taken any antibiotics during the 3 months prior to stool collection. However, among the 113 patients with IBS, 69 of them reported following a treatment for IBS symptoms during the study. These treatments included laxatives (n = 13), proton pump inhibitors (n = 25), pre/probiotics (n = 6) (Supplementary Table S2), and other medications, such as anti-depressants or anxiolytic drugs (n = 29). Since medications could have an effect, we explored their potential role on modulating the gut microbiome. For this, we re-analyzed the whole dataset, on the one hand, discarding the patients who were under treatment that could affect the microbiome composition and on the other hand, grouping them according to the treatment type that could directly affect the microbial composition (laxative, proton pump inhibitors, or probiotics/prebiotics) or indirectly (anti-depressants or anxiolytic drugs) affect the microbial composition. We were unable to take into account the effect of other drugs, since the number of patients for each of the drugs was too small and did not allow statistical analysis.

**Figure 5:**
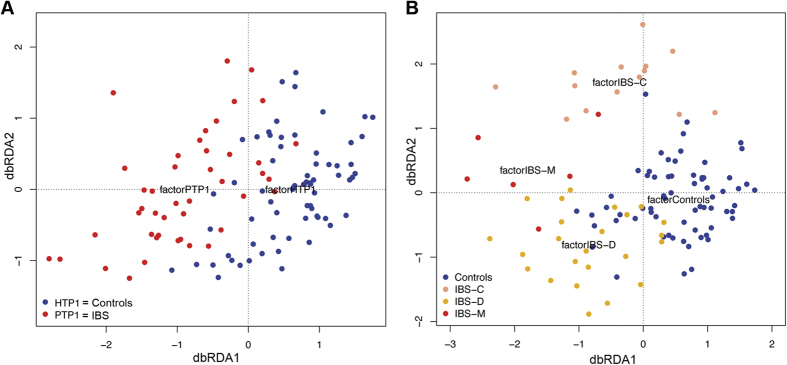
Unweighted UniFrac data redundancy analysis (dbRDA) on the first time point samples constrained by (**A**) the controls and IBS patients, and constrained by (**B**) the four groups of participants: controls (n = 66), IBS-C (n = 14), IBS-D (n = 24) and IBS-M (n = 6), discarding patients under treatment.

Removing patients who were under treatment that could affect the gut microbiome composition such as laxative, proton pump inhibitors, or probiotics/prebiotics, we re-analyzed a cohort of 73 patients (25 patients with IBS-C, 35 patients with IBS-D, 13 patients with IBS-M). Alpha-diversity analysis using the Chao1 index showed that IBS patients without treatment and without classification in subtypes continued to present a lower diversity of microbes compared to healthy controls (P = 0.002) (Supplementary Figure S3). However, significance was not reached when comparing healthy controls with each of the IBS subtypes and only IBS-D showed a trend towards a lower diversity (P = 0.09). Stability analysis also confirmed that neither the microbiome of healthy controls nor that of the IBS patients as a whole group or as subtypes differed significantly over the one-month sampling period. Distance-based redundancy analysis showed that, although only 3% of the data explained the variation, the microbiome of patients still clustered separately on the basis of their subtypes and also separately from that of the healthy controls (P = 0.001) (Fig. 5). Also, as shown in the data without discarding patients under treatment, we confirmed that Erysipelotrichaceae was in lower relative abundance in patients (P = 0.001; q = 0.04). Ruminococcaceae was also found in lower relative abundance in patients, although the difference was not significant (P = 0.03; q = 0.4). Interestingly, Methanobacteriaceae was in lower abundance (10 fold) in patients with IBS-D and IBS-M compared to healthy controls (P = 0.004; q = 0.05), which was not observed when the patients under treatment were not discarded for the analysis.

**Figure 6:**
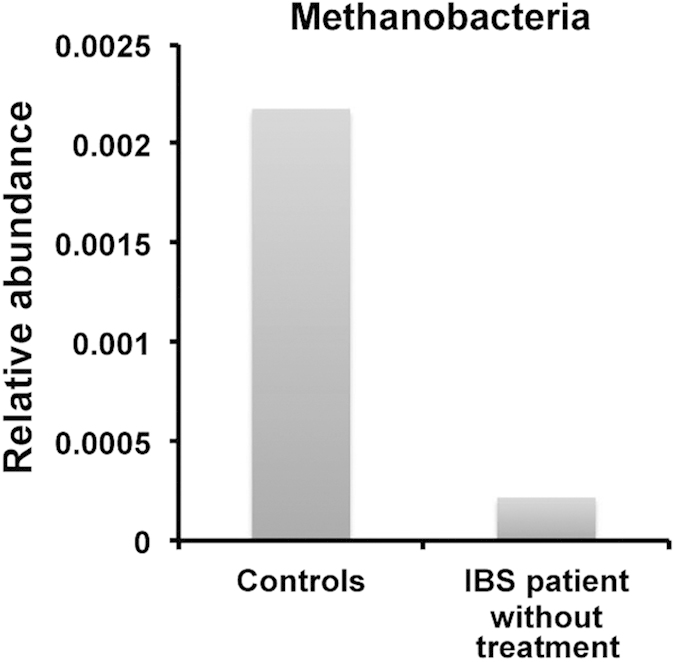
Methanobacteria from the Euryarchaeota phylum is enriched in controls (n = 66) compared to IBS patients (n = 44) who did not follow any medical treatment (Kruskal Wallis test, P = 0.005, q = 0.05).

Among the medications, we uncovered a significant effect only for the proton pump inhibitors. Indeed, patients taking proton pump inhibitors such as omeprazole (n = 25), during the study presented a 37-fold higher proportion of Pasteurellales (P = 0.002, q = 0.05), a Gammaproteobacteria group, compared to those who did not receive any treatment. At the genus level, *Haemophilus* (belonging to Pasteurellaceae) maintained this effect (P = 0.001, q = 0.08). When we compared controls (n = 66) with patients without treatment (n = 44), Methanobacteriales at the class level was also enriched in healthy controls by 10-fold (P = 0.005; q = 0.05) (Fig. 6). This group of microbes remains significantly in higher abundance in controls when compared with IBS-D patients without treatment (n = 24; P = 0.0019; q = 0.038). In the same group of patients, microbes from the Erysipelotrichi also showed a tendency of decrease (P = 0.0019; q = 0.13). IBS-C patients without treatment (n = 14) presented a higher level of Methanomassiliicoccaceae, belonging to methanogenic Archeae (P = 0.0001; q = 0.015). No significant differences were found between healthy subjects and IBS-M patients (n = 6), probably due to a too small sample size.

### Stability of the microbiota of IBS patients

Previous studies have reported the instability of the gut microbiome of IBS patients as a signature of the disease. Comparing fecal samples from patients (the whole cohort (n = 77) or without patients under treatment (n = 47)) taken one month apart, we did not detect significant differences in the microbial composition between the two time points for the IBS subtypes and as for the 22 healthy controls and no changes in microbiome associated with changes in symptoms, suggesting a stable gut microbiota over a relatively short time, which is in agreement with a previous comprehensive study by Faith *et al.*^22^. Indeed, in a previous work, we showed that instability of the gut microbiota was only conditioned by a challenge with diet enriched in fibers^23^.

### Correlation with symptoms

Taking into account only patients who did not take any medication (n = 44), we examined the correlation between microbial composition and IBS symptoms, such as sensation of flatulence and abdominal pain. Subjective sensations of flatulence are defined as anal gas evacuation, abdominal bloating (pressure/fullness), abdominal distension (girth increment), borborygmi and abdominal discomfort/pain, as described in our previous work^23^. The highest level of sensation of flatulence (level 6 in a scale of 0 to 6) was positively correlated with three OTUs from Lachnospiraceae, one belonging to the *Blautia* genus (r from 0.40 to 0.48, P < 0.006). The highest level of abdominal pain (level 6 in a scale of 0 to 6) was moderately and positively correlated with three bacterial genera, specifically *Bacteroides* (r = 0.46, P = 0.002) and *Ruminoccocus* (r = 0.42, P = 0.004), and an unknown Barnesiellaceae (r = 0.30, P = 0.041). Abdominal pain was also moderately and negatively correlated with three other genera, namely *Prevotella* (r = −0.44, P = 0.003) and *Catenibacterium* (r = −0.35, P = 0.019), a genus from the Erysipelotrichaceae family. The latter was also observed in a higher proportion in healthy controls compared to IBS patients.

## Discussion

To explore the gut microbiota in IBS, we compared the microbial communities of IBS patients with those of healthy controls using the 16S gene survey and Illumina technology. Our findings revealed that IBS patients based on their subtype clustered separately from healthy controls at a global level. Indeed, at various phylogenetic levels we showed that major differences were found between controls and IBS patients, and revealed differences between IBS subtypes, correlation of groups of bacteria with sensation of flatulence and abdominal pain, and an effect of medication on gut microbiota.

At the phylum level, our findings corroborate the results reported by Jalanka *et al.*^19^ but differ from those of Krogius *et al.*^6^, Rajilic *et al.*^13^ and Jeffery *et al.*^18^ who related higher Firmicutes and lower Bacteroidetes in IBS patients. The differences in the proportion of Gram-positive bacteria (Firmicutes) versus Gram-negative bacteria (Bacteroidetes) could be related to various factors, such as small sample size, an absence of mechanical disruption of the microbial cell wall during the DNA extraction procedure, and the DNA extraction method itself or the sequencing platform, as demonstrated by Lozupone *et al.*^24^ and by our recent work^25^. In this previous study, by freezing the fecal samples immediately after collection, using mechanical disruption during DNA extraction, performing deep sequencing using Illumina technology, and normalizing the sequences per sample, we optimized all the steps from stool collection to sequence analysis. Moreover, we previously showed that the percentage of water typically found in diarrheic samples does not affect the microbial composition of samples from the same subjects^25^.

Our results indicate that dysbiosis in IBS-D involved dominant microbial groups such as Ruminococcaceae and unknown Clostridiales, as well as much less dominant ones such as Erysipelotrichaceae and Methanobacteriaceae. Regarding Methanobacteriaceae (Archaebacteria), our finding is in line with previous studies showing a lower count of methanogens in IBS patients^13^,^17^. Interestingly, methane levels were reported to be higher in patients with slow-transit constipation compared with normal-transit constipation and non-constipated controls^26^,^27^. Our results, showing that only patients with a constipation phenotype presented higher abundance of methanogenic Archaea, confirmed a link between low transit time and methane production capacity. In the colon, methanogens and sulfate- reducing bacteria are the primary hydrogen-consuming microbes, converting this gas into methane or hydrogen sulfide, respectively^28^. However, we did not find any increase in sulfate-reducing bacteria associated with the decrease in methanogens. Furthermore, a recent metatranscriptomic study^29^ showed that *Methanobrevibacter smithii* tends to be highly transcriptionally active relative to other species in the gut, suggesting that IBS patients compared to healthy subjects lack the functions for hydrogen removal.

Regarding Ruminococcaceae, unknown Clostridiales and Erysipelotrichaceae, known as being butyrate-producing bacteria, our results suggest that a reduction of these bacteria could decrease availability of butyrate and therefore to increase the epithelial permeability, which has been previously associated with patients with IBS-D^30^. An impaired epithelial barrier function has been proposed as a potential mechanism causing passage of microbes or their products through the barrier to other body sites, which might affect symptoms through interaction with immune and nerve cells in the gut wall.

We did not find significant differences between healthy controls and IBS-C patients. Thus, our results did not confirm those obtained previously showing a higher abundance of *Veillonella* and *Ruminococcus* in this IBS subtype^7^,^8^,^9^,^10^,^12^,^13^, but showed that a group of IBS patients may harbour normal-like microbiota. Altogether, this finding suggests that any attempt to modulate the gut microbiome composition of IBS patients should take into account that all IBS patients may not respond equally to the treatment. On the basis of our results, we propose a new monitoring approach for at least two of the IBS subtypes, as a combination of four genera (an unknown Ruminococcaceae, an unknown Christensenellaceae, *Akkermansia* and *Methanobrevibacter*), which may have the capacity to discriminate healthy controls and IBS-C patients from IBS-M and IBS-D patients.

To our knowledge, our study is the first to take into account the effect of medication and proposes a cohort size large enough to perform analyses taking into account patients under treatments, which could be a confounding factor for microbiome analysis. Indeed, removing patients receiving a treatment allowed us to uncover a lower relative abundance of Methanobacteriales in IBS-D and IBS-M but a higher relative abundance of Methanomassiliicoccaceae in IBS-C. Furthermore, our results indicate that patients taking proton pump inhibitors show a significant increase in *Haemophilus*. This genus belongs to the Pasteurellaceae family and the Gammaproteobacteria class. A higher count of Gammaproteobacteria has also been reported by Krogius *et al.*^6^ and Saulnier *et al.*^7^, but it has not been associated with the administration of proton pump inhibitors. Our result is also in line with the findings of a previous study in which the authors isolated more *Haemophilus* sp. by culture from subjects under omeprazole treatment^31^. The potential adverse effects of this medication should, therefore, be addressed in greater depth in future studies. Finally, our study revealed a group of bacteria that correlates with IBS symptoms. Indeed, sensation of flatulence correlated with a high relative abundance of Lachnospiraceae and *Blautia*, while abdominal pain correlated with a high relative abundance of *Bacteroides* and *Ruminoccocus*, a low relative abundance of *Prevotella* and *Catenibacterium* and a genus belonging to Erysipelotrichaceae. Interestingly, the association of a high abundance of *Ruminoccocus* with severity of abdominal symptoms is in agreement with several studies^7^,^8^,^12^,^13^, thus confirming that this genus is involved in abdominal discomfort.

Although none of the participants underwent a strict diet intervention such as low fermentable oligo-, di-, mono-saccharides and polyols (FODMAP) diet, our study did not include a specific questionnaire regarding participants' type of diet. We are aware that, as for treatment, diet could act as another confounding variable that may affect the microbiome composition. However, previous works have shown that, unless an extreme switch in diet, short-term interventions have only a modest effect on the microbiome composition^32^,^33^,^34^. Our recommendation for future studies would be to collect diet information such as type of excluded food or type of diet intervention if any.

**Figure 7:**
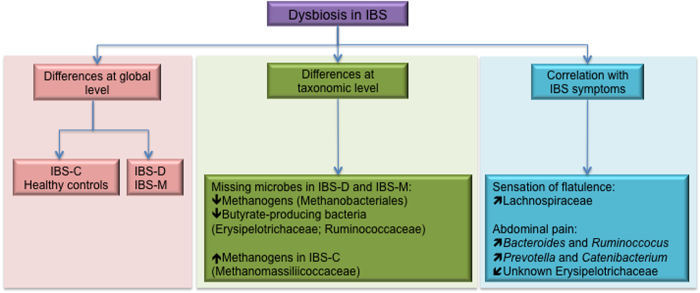
Summary of the findings of this study. 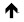
= Higher abundance of; 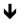
= Lower abundance of; 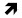
= positive correlation with; 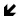
= negative correlation with.

In conclusion, our study proposes a comprehensive characterization of the microbiome in IBS, as summarized in Fig. 7. Our results showed that each IBS subtype can be differentiated from healthy controls. Patients with diarrheic phenotype shared more common microbial profiles. In contrast, IBS patients with constipation presented a microbiome similar to healthy controls, but with one exception, they harboured a greater abundance of methanogenic Archaea. The lower microbial diversity in patients, in particular in the diarrheic phenotype, could be explained by the loss of a few groups of microbes, such as methanogens, which are involved in H2 removal, and loss of butyrate-producing bacteria, such as Erysipelotrichaceae. In this regard, the development of a method to reduce the microbe populations involved in low transit, such as methanogens in IBS-C, to increase those missing in IBS-D and IBS-M, or to increase those negatively correlated with abdominal pain, by bacteriotherapy for instance, may offer a therapeutic strategy for the management of these patients. Furthermore, our study stresses the need to include patients not receiving treatment and psychological data in microbiome-IBS studies.

## Material and Methods

### Participants

All experiments were performed in accordance to ethical guidelines. The subjects gave written informed consent to participate in this study. The protocol was submitted and approved by the local Ethical Committee of the University Hospital Vall d’Hebron (Barcelona, Spain). Outpatients fulfilling Rome III criteria for IBS and healthy subjects were prospectively enrolled over 2.5 years into the study^2^. All participants filled out a clinical questionnaire specifying stool form according to a Bristol stool scale, symptoms and medications. The Rome III criteria include recurrent abdominal pain or discomfort at least 3 days per month in the last 3 months with symptom onset at least 6 months prior to diagnosis associated with 2 or more of the following: improvement with defecation; onset associated with a change in frequency of stool; onset associated with a change in form of stool. IBS was subtyped by predominant stool pattern for each patient. IBS with constipation (IBS-C) was defined by hard or lumpy stools ≥25% and loose (mushy) or watery stools ≤25% of bowel movements. IBS with diarrhea (IBS-D) was defined by loose (mushy) or watery stools ≥25% and hard or lumpy stool ≤25% of bowel movements. Mixed IBS (IBS-M) was defined by hard or lumpy stools ≥25% and loose (mushy) or watery stools ≥25% of bowel movements.

Individuals (non-related (91%) and among the research and hospital environment (9%)) without gastrointestinal symptoms and matching age, gender and BMI with patients were included as healthy controls. The participants did not fill out a specific questionnaire on their diet habit, but none of them (healthy controls and patients) had a coeliac disease and none of them were following an extreme diet intervention such as low FODMAP diet. Information on medications that could have a direct impact on gut microbiota is provided in Supplementary Table S2. Participants were included if they had not taken antibiotics in the previous three months. None of the healthy controls took any medication during the study. All subjects gave written informed consent to participate in this study. The protocol was submitted and approved by the local Ethical Committee of the University Hospital Vall d’Hebron (Barcelona, Spain). Information about group size, age, gender and BMI is given in Table 1.

### Sample collection and genomic DNA extraction

Fecal samples were collected from the 179 participants (66 healthy controls and 113 IBS patients). In order to study the variability of the microbiome over time, 22 healthy controls and 72 IBS patients (IBS-D, n = 33; IBS-C, n = 26 and IBS-M, n = 13) provided a second stool sample one month after the first. After collection and homogenization, samples were immediately frozen by the participants in their home freezer at −20 °C and later brought to the laboratory in a freezer pack, where they were stored at −80 °C. A frozen aliquot (250 mg) of each sample was suspended in 250 μl of guanidine thiocyanate, 0.1 M Tris (pH 7.5), 40 μl of 10% N-lauroyl sarcosine and 500 μl 5% N-lauroyl sarcosine. DNA was extracted by mechanical disruption of the microbial cells with beads, and recovery of nucleic acids from clear lysates was achieved by alcohol precipitation, as previously described^25^,^35^. An equivalent of 1 mg of each sample was used for DNA quantification using a NanoDrop ND-1000 Spectrophotometer (Nucliber). DNA integrity was examined by micro-capillary electrophoresis using an Agilent 2100 Bioanalyzer with the DNA 12,000 kit, which resolves the distribution of double-stranded DNA fragments up to 17,000 bp in length.

### High-throughput DNA sequencing

For profiling microbiome composition, the hyper-variable region (V4) of the bacterial and archaeal 16S rRNA gene was amplified by PCR. On the basis of our analysis done using Primer Prospector software^36^, the V4 primer pairs used in this study were expected to amplify almost 100% of the bacterial and archaeal domains. The 5’ ends of the forward (V4F_515_19: 5’- GTGCCAGCMGCCGCGGTAA -3’) and reverse (V4R_806_20: 5’- GGACTACHVGGGTWTCTAAT -3’) primers targeting the 16S gene were tagged with specific sequences as follows: 5’-{AATGATACGGCGACCACCGAGATCTACACTATGGTAATTGT} {GTGCCAGCMGCCGCGGTAA}-3’ and 5’-{CAAGCAGAAGACGGCATACGAGAT} {Golay barcode} {AGTCAGTCAGCC} {GGACTACHVGGGTWTCTAAT}-3’. Multiplex identifiers, known as Golay codes had 12 bases and were specified downstream of the reverse primer sequence (V4R_806_20)^37^,^38^.

Standard PCR (0.75 units of Taq polymerase (Roche) and 20 pmol/μL of the forward and reverse primers) was run in a Mastercycler gradient (Eppendorf) at 94 °C for 3 min, followed by 35 cycles of 94 °C for 45 sec, 56 °C for 60 sec, 72 °C for 90 sec, and a final cycle of 72 °C for 10 min. Amplicons were first purified using the QIAquick PCR Purification Kit (Qiagen, Barcelona, Spain), quantified using a NanoDrop ND-1000 Spectrophotometer (Nucliber), and then pooled in equal concentration. The pooled amplicons (2 nM) were then subjected to sequencing using Illumina MiSeq technology at the technical support unit of the Autonomous University of Barcelona (UAB, Spain) following standard Illumina platform protocols.

### Sequence data analysis

We loaded the raw sequences into the QIIME 1.8.0 pipeline for analysis, as described by Navas-Molina *et al.*^37^. The first step was to filter out quality sequence reads by applying default settings and a minimum acceptable Phred score of 20. Correct primer and proper barcode sequences were also checked. After filtering, from 290 fecal samples we obtained a total of 12,494,196 high-quality sequences with a read length ranging from 237 to 307 nucleotides; 73% of the reads had a length of 306 nucleotides.

We used the UCLUST^39^ algorithm to cluster similar filtered sequences into OTUs based on a 97% similarity threshold. Then, we identified and removed chimeric sequences using ChimeraSlayer^40^. Since each OTU can comprise many related sequences, we picked a representative sequence from each one. Representative sequences were aligned using PyNAST against Greengenes template alignment (gg_13_8 release), and taxonomy was assigned to the detected OTUs using the basic local alignment search tool (BLAST) reference database and the Greengenes taxonomy-mapping file. The script make_phylogeny.py was used to create phylogenetic trees using the FastTree program^41^. To correctly define species richness for the analysis of between-sample diversity, known as beta diversity, the OTU table was rarefied at 16,800 sequences per sample. Rarefaction is used to overcome cases in which read counts were not similar in numbers between samples. Next, the OTU table was split into the different groups (Diarrhea (IBS-D), Constipation (IBS-C), Mixed (IBS-M) IBS, and Healthy Controls (HC)). In order to avoid false positive taxa, OTUs that did not represent at least 0.2% of sequences for any given sample were removed from the resulting OTU table. The summarize taxa feature was used to classify taxa from the Domain to the Species level. To provide community alpha diversity estimates, we calculated Chao1^42^. To calculate between-sample diversity, weighted and unweighted Unifrac metrics were applied to build phylogenetic distance matrices, which were then used to construct hierarchical cluster trees using unweighted pair group method with arithmetic mean (UPGMA) and PCoA representations. Finally, distance-based redundancy analyses were performed using the eigenvalues obtained in the PCoA.

### Statistical and correlation analyses

Statistical analyses were carried out in QIIME. To work with normalized data, we analyzed an equal number of sequences from both healthy and patient groups. The Shapiro-Wilk test^43^ was used to check the normality of data distribution. The Kruskal Wallis one way analysis of variance^44^, a non-parametric test, was used to compare the mean number of sequences of the groups, i.e. that of different groups of patients based on distinct parameters with that of healthy subjects, in various taxonomic levels. Since we used nonparametric correlations, significance was determined through permutations. The analysis provided false discovery rate (FDR) corrected P-values (q).

Statistical significance of observed differences between sample groups was measured by the t-test, with P-values < 0.05 considered significant for all tests. Non-parametric Spearman's correlation coefficient was used to calculate possible relationships among microbial genera and variables in clinical data such as abdominal pain and sensation of flatulence.

## Additional Information

**How to cite this article**: Pozuelo, M. *et al.* Reduction of butyrate- and methane-producing microorganisms in patients with Irritable Bowel Syndrome. *Sci. Rep.* **5**, 12693; doi: 10.1038/srep12693 (2015).

**Accession codes**: The sequences have been upload to NCBI-SRA with the accession number PRJNA268708.

## Supplementary Material

Supplementary Information

## Figures and Tables

**Table 1:** Clinical data from healthy controls and patients with IBS or other functional disorders.

	N	Second Time Point n	Average Age	Gender	BMI^a^
Healthy Controls	66	22	37.6 (18–63, SD = 13)	40F/26M	23.7 (SD = 3.4)
IBS^b^	113	72	42.6 (20–86, SD = 13)	80F/33M	23.7 (SD = 4)
IBS-D^c^	54	33	41.9 (SD = 13)	29F/25M	25 (SD = 4.6)
IBS-C^d^	32	26	39.4 (SD = 10.8)	31F/2M	23.3 (SD = 3.8)
IBS-M^e^	27	13	48.2 (SD = 16.4)	32F/8M	23.9 (SD = 3.6)

^a^Body Mass Index.

^b^Irritable Bowel Syndrome.

^c^Irritable Bowel Syndrome with diarrhea.

^d^Irritable Bowel Syndrome with constipation.

^e^Mixed Irritable Bowel Syndrome.
