# Supplementary material for: Reduction of butyrate- and methane-producing microorganisms in patients with Irritable Bowel Syndrome
Source: Sci Rep. 2015 Aug 4;5:12693. doi: 10.1038/srep12693 (PMC4523847; doi:10.1038/srep12693)
Supplement: Supplementary Information [file srep12693-s1.pdf]

Reduction of butyrate- and methane-producing microorganisms in patients with Irritable Bowel Syndrome

Authors: Marta Pozuelo[#],[1] Suchita Panda[#],[1] Alba Santiago, [1] Sara Mendez, [2] Anna Accarino, [2,3] Javier Santos, [1,2,3] Francisco Guarner, [1,2,3] Fernando Azpiroz, [1,2,3] and Chaysavanh Manichanh*[1,3]

[#]**Authors share co-first authorship**

[1]Digestive System Research Unit, Vall d'Hebron Research Institute, Passeig Vall d'Hebron 119-129, Barcelona 08035, Spain

[2]Digestive Unit, University Hospital Vall d'Hebron, Passeig Vall d'Hebron 119-129, Barcelona 08035, Spain

[3]Centro de Investigacion Biomedica en Red en el Área tematica de Enfermedades Heptaticas y Digestivas, CIBERehd, Instituto de Salud Carlos III, Madrid, Spain

Supplementary Table S1. Summary of studies on gut microbiota and IBS using 16S rRNA gene survey.

Year	IBS subtypes N and Healthy controls N	Gender in IBS	Type of study	Treatment during the study	Sample type	Technique used	Sequence depth for sequencing techniques	Results	References
**2005**	IBS-D 12; IBS-C 9; IBS-A 6; HC 22	ND	Longitudinal 3 months interval	Regular IBS symptoms	Feces	qPCR 16S rRNA	ND	Lower amount of *Lactobacillus* in IBS-D; higher amount of *Veillonella* in IBS-C; higher *Ruminococcus productus* - *Clostridium coccoides* in IBS; lower *Bifidobacterium catenulatum* in IBS	1
**2005**	IBS-D 12; IBS-C 9; IBS-A 5; HC 25	19 females; 7 males	Longitudinal 0, 3 and 6 months	IBS symptoms, antibiotics	Feces	PCR-DGGE	ND	Increase of coliforms; increase of aerobe/anaerobe ratio, temporal instability in IBS but explained by antibiotics intake	2
**2006**	IBS-D 7; IBS-C 6; IBS-A 3; HC 16	11 females; 5 males	Longitudinal 0, 6 months	ND	Feces	PCR-DGGE; quantitative hybridization-based technique, transcript analysis	ND	*Clostridium coccoides* - Eubacterium rectale group lower in IBS-C; higher instability in IBS	3
**2007**	IBS-D 10; IBS-C 8; IBS-M 6; HC 23	19 females; 5 males	Cross-sectional	ND	Feces	Cloning-sequencing and qPCR	3,753 for all samples	Different bacterial structure in IBS compared to HC; differences in genera *Coprococcus*, *Collinsella*, and *Coprobacillus* between IBS and HC	4
**2009**	IBS-D 14; IBS-C 11; IBS-A 16; HC 26	29 females; 12 males	Cross-sectional	No medication that could influence microbial composition	Feces; duodenal mucosa	FISH; qPCR	ND	Decrease of bifidobacteria in faeces and *Bifidobacterium catenulatum* in feces and duodenal samples of IBS	5
**2009**	IBS-D 10; HC 23	6 females; 4 males	Cross-sectional	ND	Feces	%G+C-based profiling and fractioning combined with 16S rRNA cloning/sequencing ; qPCR	3,267 sequencing for all samples	IBS-D is enriched in Proteobacteria and Firmicutes Lachnospiraceae but reduced in Actinobacteria and Bacteroidetes	6
**2009**	IBS-D 8; IBS-C 8; IBS-M 4; HC 15	14 females; 6 males	Longitudinal 0, 3, 6 months	IBS medication mainly commercial fiber analogs, laxatives, or antidiarrhoeals	Feces	qPCR	ND	Microbiota of the IBS-D patients differed from other sample groups *Clostridium thermosuccinogenes*-like, *Ruminococcus torques*-like; a *Ruminococcus bromii* -like phylotype was associated with IBS-C patients in comparison to controls	7
**2010**	IBS-D 10; HC 10	7 females; 3 males	Cross-sectional	No probiotic	Feces; mucosal biopsy	qPCR; culture	ND	Decreased concentration of aerobic bacteria in IBS-D; increase in *Lactobacillus* in IBS-D	8
**2010**	IBS 47; HC 33	47 female	Cross-sectional	No medication	Feces; colonic biopsies	DGGE	ND	Different microbial community in feces and colonic biopsies; difference in the gut microbiota between feces from IBS and HC	9

**2010**	IBS 44	33 females; 11 males	Cross-sectional	No medication	Feces	qPCR	ND	*Ruminococcus torques*-like was associated with severity of bowel symptoms	10
**2010**	IBS-D 8; IBS-C 11; IBS-M 7; HC 26	13 females; 13 males	Cross-sectional	ND	Feces	qPCR; culture; organic acids analysis HPLC; quantification of bowel gas X-ray film	ND	Quantity of gas similar in IBS and HC; high acetic and propionic acid correlate with worse GI symptoms; high count of *Veillonella* and *Lactobacillus* in IBS	11
**2011**	IBS-D 16; HC 21	12 females; 4 males	Cross-sectional		Luminal and mucosal samples	T-RFLP	ND	Lower microbial diversity in IBS-D	12
**2011**	IBS-D 13; IBS-C 11; IBS-A 13; HC 12	ND	Cross-sectional	No medication	Feces; mucosa-associated small intestinal	16S DGGE; qPCR; cloning sequencing	ND	*Pseudomonas aeruginosa* more abundant in feces and small intestine in IBS	13
**2011**	IBS-D 25; IBS-C 18; IBS-A 19; HC 46	57 females; 5 males	Cross-sectional	ND	Feces	qPCR; 16S phylogenetic microarray 129 genus-like groups	ND	Increased Firmicutes / Bacteroidetes ratio in IBS; increased Dorea, *Ruminococcus* and *Clostridium*, but decreased *Bifidobacterium* and methanogens in IBS; decreased *Faecalibacterium* in IBS-A and IBS-C; more Archaea in HC	14
**2011**	IBS-D 1; IBS-C 13; IBS-U 7; HC 22	8 females; 14 males pediatrics 7-12y	Longitudinal 6 months	ND	Feces	16S pyrosequencing; phylogenetic microarray 8000 species	54,200 reads per sample	Higher abundance of Gammaproteobacteria *Haemophilus parainfluenzae* and *Ruminococcus* in IBS; pain correlates with *Alistipes* genus	15
**2012**	IBS-D 23; HC 23	17 females; 6 males	Cross-sectional	No probiotics 2 months prior to study; no anti-inflammatories.	Feces	16S pyrosequencing	8,232 reads V1-3 and 6,591 reads V6 per sample	Higher proportion of Enterobacteriaceae, lower *Faecalibacterium* and lower diversity in IBS-D	16
**2012**	IBS-C 14; HC 12	14 females	Cross-sectional	No laxatives, antidiarrhoeal, antispasmodi, analgesic	Feces	Culture of anaerobes; FISH	ND	Lower numbers of lactate-producing, lactate-utilising bacteria, H2-consuming populations, methanogens and reductive acetogens, but higher number of lactate- and H2-utilising sulphate-reducing bacteria in IBS-C	17
**2012**	IBS-D 14; HC 18	3 females; 11 males	Cross-sectional	No medication	Feces	Fecal BA profiles; q-PCR	ND	Bile acids higher in IBS-D and correlated with stool consistency and frequency; increase of *Escherichia coli* and a significant decrease of leptum and *Bifidobacterium* in IBS-D	18
**2012**	IBS-D 13; IBS-C 3; HC 9	ND	Cross-sectional	ND	Feces; Mucosal biopsies	16S pyrosequencing	268,000 non-chimeric reads	Less diversity in IBS; larger differences in the microbiota composition between biopsies and feces than between patients and controls	19
**2012**	IBS-D 15; IBS-C 10; IBS-A 12;	26 females; 11 males	Cross-sectional	No medication	Feces	16S pyrosequencing	30,000 reads per sample	Associations detected between microbiota composition and clinical or physiological phenotypes; two subgroups of IBS: normal-	20

	HC 20							like and abnormal-like. The latter showed increased Firmicutes / Bacteroidetes ratio.	
**2012**	IBS-D 27; IBS-C 20; HC 26	ND	Cross-sectional	ND	Rectal biopsies	FISH	ND	Greater numbers of total mucosa-associated bacteria in patients; greater *Bacteroides* and *Eubacterium rectale - Clostridium coccoides* in IBS; bifidobacteria were lower in the IBS-D group than in the IBS-C group and controls	21
**2012**	IBS-D 22; HC 22	12 females; 10 males Pediatrics 12.6 y	Cross-sectional	No medication	Feces	16S Phylogenetic microarray 775 phylotypes; 16S pyrosequencing; FISH; qPCR	A total of 13,882 sequence reads from the four chosen samples	Increased levels of *Veillonella*, *Prevotella*, *Lactobacillus*, and *Parasporobacterium* in IBS-D; No difference in alpha-diversity between IBS and HC; larger phylotype core set in HC than in IBS-D; *Bifidobacterium* and Verrucomicrobium less abundant in IBS-D; positive correlations between *Veillonella* and both *Haemophilus* and *Streptococcus*, between *Anaerovorax* and *Verrucomicrobium* and between *Tannerella* and *Anaerophaga*	22
**2013**	IBS-D 2; HC 1	2 females	Longitudinal: less diarrhoea days 1, 3, and 7 in Patient 1; day 35 in Patient 2; severe diarrhoea days 14, 28, 37, and 42 in Patient 1; days 3 and 28 in Patient 2.	Hypertension, arthrosis, osteoporosis for patient 1; bowel spasms and contraceptives for patient 2	Feces	Metagenomics and metatranscriptomics by pyrosequencing	2,013,366 reads for metagenomics 7 samples; 1,729,416 reads for metatranscriptomic 32 samples	Higher temporal instability in the fraction of active microbiota related to the IBS condition and fluctuating symptoms	23
**2013**	PI-IBS 11; PI-nonBD 12; PI-BD 11; IBS-D 12 HC 11	29 females; 17 males	Cross-sectional	ND	Biopsy; feces	Host gene expression by microarray; phylogenetic microarray 130 genus-like groups	ND	27 genus-like groups IMD separating HC and patients; more uncultured Clostridia in HC; more Bacteroidetes members in patients; correlation between IMD and host genes involved in epithelial barrier functions in IBS	24

N = Number of participants
ND = No data
HC = Healthy controls
IBS-D = Diarrhea-predominant IBS
IBS-C = Constipation-predominant IBS
IBS-M = Mixed IBS
IBS-A = Alternating IBS
IBS-U = unsubtyped IBS
PI-IBS = Post-infectious IBS
PI-BD = Persistent bowel dysfunction
PI-non-BD = no bowel dysfunction
FISH = Fluorescent in situ hybridization
IMD = Index of Microbial Dysbiosis
16S = 16S rRNA gene
DGGE = Denaturing Gradient Gel Electrophoresis

REFERENCES

1. Malinen E, Rinttila T, Kajander K *et al.* Analysis of the fecal microbiota of irritable bowel syndrome patients and healthy controls with real-time PCR. Am J Gastroenterol 2005;100:373-82.
2. Matto J, Maunuksela L, Kajander K *et al.* Composition and temporal stability of gastrointestinal microbiota in irritable bowel syndrome--a longitudinal study in IBS and control subjects. FEMS Immunol Med Microbiol 2005;43:213-22.
3. Maukonen J, Satokari R, Matto J *et al.* Prevalence and temporal stability of selected clostridial groups in irritable bowel syndrome in relation to predominant faecal bacteria. J Med Microbiol 2006;55:625-33.
4. Kassinen A, Krogius-Kurikka L, Makivuokko H *et al.* The fecal microbiota of irritable bowel syndrome patients differs significantly from that of healthy subjects. Gastroenterology 2007;133:24-33.
5. Kerckhoffs AP, Samsom M, van der Rest ME *et al.* Lower Bifidobacteria counts in both duodenal mucosa-associated and fecal microbiota in irritable bowel syndrome patients. World J Gastroenterol 2009;15:2887-92.
6. Krogius-Kurikka L, Lyra A, Malinen E *et al.* Microbial community analysis reveals high level phylogenetic alterations in the overall gastrointestinal microbiota of diarrhoea-predominant irritable bowel syndrome sufferers. BMC Gastroenterol 2009;9:95,230X-9-95.
7. Lyra A, Rinttila T, Nikkila J *et al.* Diarrhoea-predominant irritable bowel syndrome distinguishable by 16S rRNA gene phylotype quantification. World J Gastroenterol 2009;15:5936-45.
8. Carroll IM, Chang YH, Park J *et al.* Luminal and mucosal-associated intestinal microbiota in patients with diarrhea-predominant irritable bowel syndrome. Gut Pathog 2010;2:19,4749-2-19.
9. Codling C, O'Mahony L, Shanahan F *et al.* A molecular analysis of fecal and mucosal bacterial communities in irritable bowel syndrome. Dig Dis Sci 2010;55:392-7.
10. Malinen E, Krogius-Kurikka L, Lyra A *et al.* Association of symptoms with gastrointestinal microbiota in irritable bowel syndrome. World J Gastroenterol 2010;16:4532-40.
11. Tana C, Umesaki Y, Imaoka A *et al.* Altered profiles of intestinal microbiota and organic acids may be the origin of symptoms in irritable bowel syndrome. Neurogastroenterol Motil 2010;22:512,9, e114-5.
12. Carroll IM, Ringel-Kulka T, Keku TO *et al.* Molecular analysis of the luminal- and mucosal-associated intestinal microbiota in diarrhea-predominant irritable bowel syndrome. Am J Physiol Gastrointest Liver Physiol 2011;301:G799-807.
13. Kerckhoffs AP, Ben-Amor K, Samsom M *et al.* Molecular analysis of faecal and duodenal samples reveals significantly higher prevalence and numbers of Pseudomonas aeruginosa in irritable bowel syndrome. J Med Microbiol 2011;60:236-45.
14. Rajilic-Stojanovic M, Biagi E, Heilig HG *et al.* Global and deep molecular analysis of microbiota signatures in fecal samples from patients with irritable bowel syndrome. Gastroenterology 2011;141:1792-801.
15. Saulnier DM, Riehle K, Mistretta TA *et al.* Gastrointestinal microbiome signatures of pediatric patients with irritable bowel syndrome. Gastroenterology 2011;141:1782-91.

16. Carroll IM, Ringel-Kulka T, Siddle JP *et al.* Alterations in composition and diversity of the intestinal microbiota in patients with diarrhea-predominant irritable bowel syndrome. Neurogastroenterol Motil 2012;24:521,30, e248.
17. Chassard C, Dapoigny M, Scott KP *et al.* Functional dysbiosis within the gut microbiota of patients with constipated-irritable bowel syndrome. Aliment Pharmacol Ther 2012;35:828-38.
18. Duboc H, Rainteau D, Rajca S *et al.* Increase in fecal primary bile acids and dysbiosis in patients with diarrhea-predominant irritable bowel syndrome. Neurogastroenterol Motil 2012;24:513,20, e246-7.
19. Durban A, Abellan JJ, Jimenez-Hernandez N *et al.* Structural alterations of faecal and mucosa-associated bacterial communities in irritable bowel syndrome. Environ Microbiol Rep 2012;4:242-7.
20. Jeffery IB, O'Toole PW, Ohman L *et al.* An irritable bowel syndrome subtype defined by species-specific alterations in faecal microbiota. Gut 2012;61:997-1006.
21. Parkes GC, Rayment NB, Hudspith BN *et al.* Distinct microbial populations exist in the mucosa-associated microbiota of sub-groups of irritable bowel syndrome. Neurogastroenterol Motil 2012;24:31-9.
22. Rigsbee L, Agans R, Shankar V *et al.* Quantitative profiling of gut microbiota of children with diarrhea-predominant irritable bowel syndrome. Am J Gastroenterol 2012;107:1740-51.
23. Durban A, Abellan JJ, Jimenez-Hernandez N *et al.* Instability of the faecal microbiota in diarrhoea-predominant irritable bowel syndrome. FEMS Microbiol Ecol 2013;86:581-9.
24. Jalanka-Tuovinen J, Salojarvi J, Salonen A *et al.* Faecal microbiota composition and host-microbe cross-talk following gastroenteritis and in postinfectious irritable bowel syndrome. Gut 2014;63:1737-45.

Supplementary Table S2. Medications that could have a direct impact on gut microbiota and food avoided by patients

#SampleID	Description	Proton pump inhibitors	Laxatives	Anti-diarrhoeics	Pre/probiotics	Food avoided by patients
**MO.01**	IBS_Diarrhoea	Omeprazole				Natural tomato paste
**MO.12**	IBS_Diarrhoea	Omeprazole				
**MO.13**	IBS_Diarrhoea	Omeprazole				Milk, yogurt, spicy food
**MO.15**	IBS_Diarrhoea				VSL3	
**MO.17**	IBS_Diarrhoea	Omeprazole	Psyllium husk			
**MO.20**	IBS_Diarrhoea		Psyllium husk			
**MO.21**	IBS_Diarrhoea	Omeprazole				Raw vegetables, coffee, fruits, eggs, cheese, spicy food
**MO.25**	IBS_Diarrhoea					
**MO.27**	IBS_Diarrhoea	Omeprazole				Legumes, soup, chocolate, coffee, lactic products, greasy food, excessive water intake
**MO.32**	IBS_Diarrhoea	Pantoprazole				
**MO.35**	IBS_Diarrhoea					Greasy food
**MO.39**	IBS_Diarrhoea	Omeprazole				Vegetables
**MO.42**	IBS_Diarrhoea					
**MO.45**	IBS_Diarrhoea					
**MO.47**	IBS_Diarrhoea					Milk, greasy food, capsicum
**MO.49**	IBS_Diarrhoea		Lactitol Monohydrate			
**MO.53**	IBS_Diarrhoea	Rabeprazole				
**MO.54**	IBS_Diarrhoea					
**MO.58**	IBS_Diarrhoea					
**MO.60**	IBS_Diarrhoea	Rabeprazole				
**MO.62**	IBS_Diarrhoea	Omeprazole				Blue fish, cucumber, melon
**MO.68**	IBS_Diarrhoea					
**MO.71**	IBS_Diarrhoea					Gluten
**MO.74**	IBS_Diarrhoea				Ferzym plus®	
**MO.82**	IBS_Diarrhoea				Probiotic	
**MO.87**	IBS_Diarrhoea					Lactic food, legumes, fizzy drinks, fried food
**MO.88**	IBS_Diarrhoea		Psyllium husk			Bread, pasta
**MO.97**	IBS_Diarrhoea					
**MO.98**	IBS_Diarrhoea	Omeprazole				
**MO.106**	IBS_Diarrhoea			Loperamide		
**MO.108**	IBS_Diarrhoea	Omeprazole				Lactic food
**MO.111**	IBS_Diarrhoea					
**MO.115**	IBS_Diarrhoea					Greasy food
**MO.120**	IBS_Diarrhoea					Flour products, potato, legumes, lactic food
**MO.06**	IBS_Constipation					
**MO.09**	IBS_Constipation					
**MO.55**	IBS_Constipation					
**MO.63**	IBS_Constipation					
**MO.64**	IBS_Constipation					
**MO.69**	IBS_Constipation					
**MO.75**	IBS_Constipation					Oil, greasy and fried food
**MO.77**	IBS_Constipation		Lactitol Monohydrate			
**MO.79**	IBS_Constipation	Omeprazole				
**MO.80**	IBS_Constipation	Omeprazole				
**MO.89**	IBS_Constipation					
**MO.92**	IBS_Constipation	Omeprazole	Psyllium husk			
**MO.96**	IBS_Constipation					
**MO.101**	IBS_Constipation				Bifilax	
**MO.104**	IBS_Constipation					
**MO.112**	IBS_Constipation	Esomeprazole				
**MO.125**	IBS_Constipation					
**MO.127**	IBS_Constipation		Psyllium husk			
**MO.04**	Mixed_IBS	Pantoprazole				
**MO.05**	Mixed_IBS					Vegetables, tomato paste, salsa
**MO.08**	Mixed_IBS	Esomeprazole				
**MO.10**	Mixed_IBS					Vegetables, skimmed milk
**MO.16**	Mixed_IBS	Omeprazole	Psyllium husk			
**MO.23**	Mixed_IBS	Omeprazole				
**MO.24**	Mixed_IBS					Chicken and eggs
**MO.28**	Mixed_IBS				Probiotic	Fried food, artichoke, capsicum, salsa, lactic food
**MO.34**	Mixed_IBS	Omeprazole	Psyllium husk			
**MO.36**	Mixed_IBS		Psyllium husk			Fried food, desserts, coffee, alcohol, salsa, fizzy drinks, lactic

					food
**MO.43**	Mixed_IBS				
**MO.48**	Mixed_IBS	Pantoprazole			
**MO.59**	Mixed_IBS		Movicol®		
**MO.65**	Mixed_IBS		Psyllium husk		
**MO.66**	Mixed_IBS		Psyllium husk		
**MO.76**	Mixed_IBS	Pantoprazole			
**MO.81**	Mixed_IBS				Milk, lactic food
**MO.109**	Mixed_IBS			Ultra levura®	
**MO.121**	Mixed_IBS	Omeprazole			
**MO.122**	Mixed_IBS				
**MO.126**	Mixed_IBS				

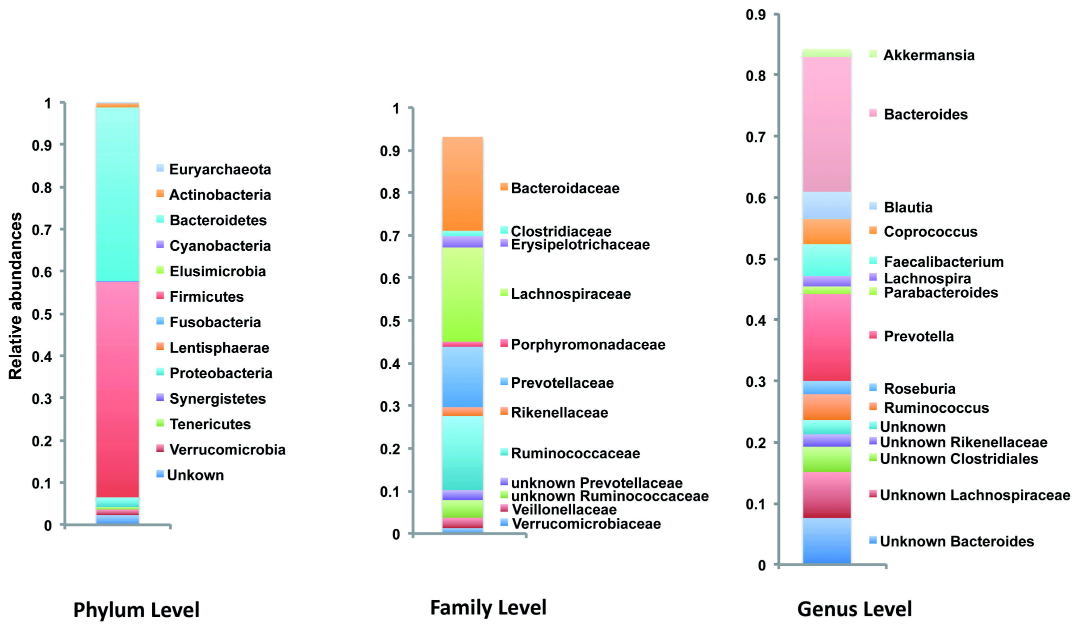

Supplementary Figure S1. Diversity of the human microbiota at various phylogenetic levels. Using the 16S rRNA sequence data of 66 healthy controls, the average relative abundance of each microbial group is represented at phylum, family, and genus level.

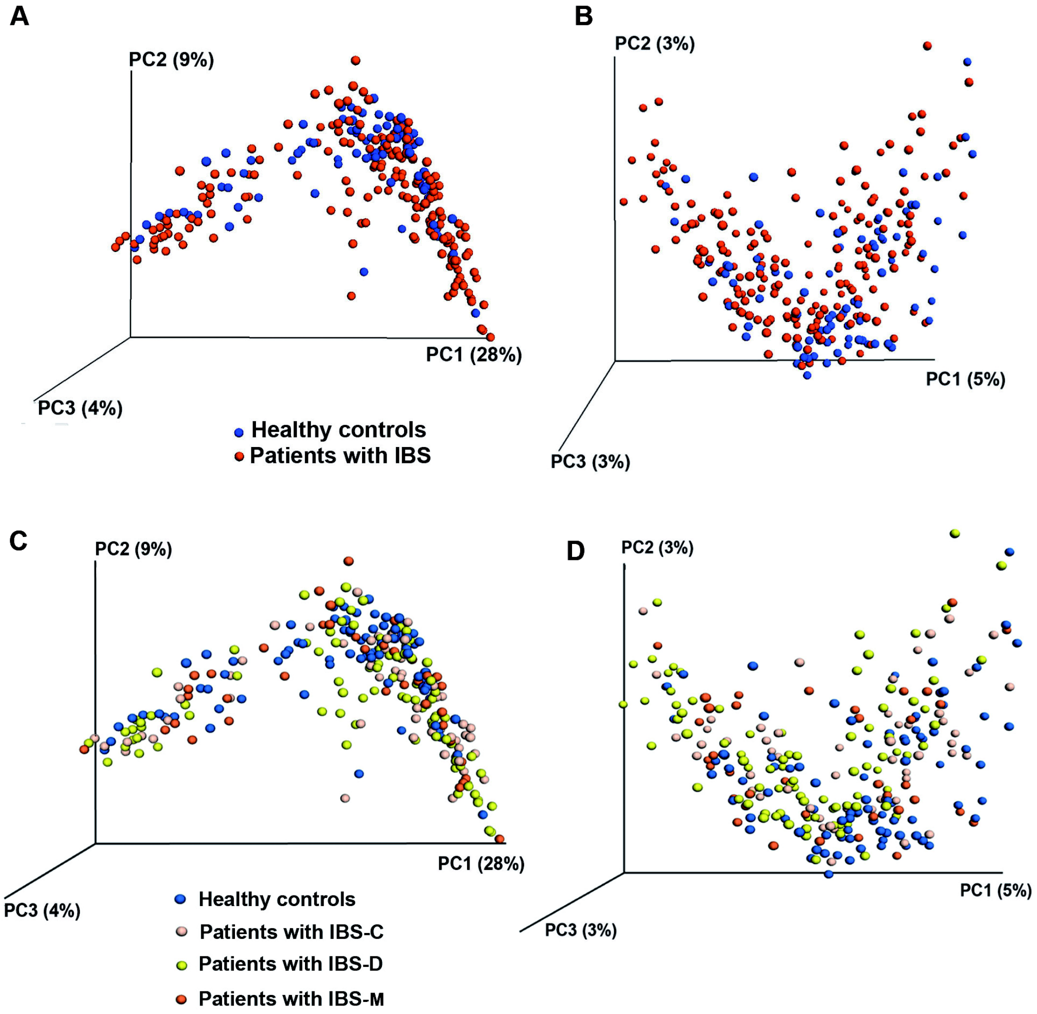

Supplementary Figure S2. Weighted UniFrac PCoA plot of the whole dataset (A), and unweighted UniFrac PCoA plot of the whole dataset (B), weighted UniFrac PCoA plot of the controls and the three IBS subtypes (C), and unweighted UniFrac PCoA plot of the controls and the three IBS subtypes (D) illustrate that samples from healthy controls and patients with IBS did not cluster separately.

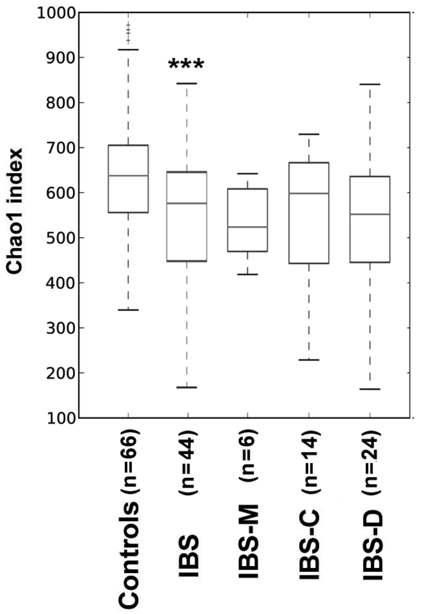

Supplementary Figure S3. The Chao1 index based on species-level OTUs was estimated for healthy controls, IBS, IBS-M, IBS-C and IBS-D patients, taking into account only participants not receiving treatment. Significance (***P=0.002) was determined by Monte Carlo permutations, a non-parametric test.
